# ECM Remodeling in Squamous Cell Carcinoma of the Aerodigestive Tract: Pathways for Cancer Dissemination and Emerging Biomarkers

**DOI:** 10.3390/cancers13112759

**Published:** 2021-06-02

**Authors:** Albina Fejza, Lucrezia Camicia, Evelina Poletto, Greta Carobolante, Maurizio Mongiat, Eva Andreuzzi

**Affiliations:** Division of Molecular Oncology, Department of Research and Diagnosis, Centro di Riferimento Oncologico di Aviano (CRO) IRCCS, 33081 Aviano, Italy; albina.fejza@cro.it (A.F.); lucricamicia@gmail.com (L.C.); evelina.poletto@cro.it (E.P.); greta.carobolante@cro.it (G.C.); mmongiat@cro.it (M.M.)

**Keywords:** squamous cell carcinoma, extracellular matrix, metastatic dissemination, tumor microenvironment

## Abstract

**Simple Summary:**

Local and distant metastasis of patients affected by squamous cell carcinoma of the upper aerodigestive tract predicts poor prognosis. In the latest years, the introduction of new therapeutic approaches, including targeted and immune therapies, has improved the overall survival. However, a large number of these patients do not benefit from these treatments. Thus, the identification of suitable prognostic and predictive biomarkers, as well as the discovery of new therapeutic targets have emerged as a crucial clinical need. In this context, the extracellular matrix represents a suitable target for the development of such therapeutic tools. In fact, the extracellular matrix is composed by complex molecules able to interact with a plethora of receptors and growth factors, thus modulating the dynamic crosstalk between cancer cells and the tumor microenvironment. In this review, we summarize the current knowledge of the role of the extracellular matrix in affecting squamous cell carcinoma growth and dissemination. Despite extracellular matrix is known to affect the development of many cancer types, only a restricted number of these molecules have been recognized to impact on squamous cell carcinoma progression. Thus, we consider that a thorough analysis of these molecules may be key to develop new potential therapeutic targets/biomarkers.

**Abstract:**

Squamous cell carcinomas (SCC) include a number of different types of tumors developing in the skin, in hollow organs, as well as the upper aerodigestive tract (UADT) including the head and neck region and the esophagus which will be dealt with in this review. These tumors are often refractory to current therapeutic approaches with poor patient outcome. The most important prognostic determinant of SCC tumors is the presence of distant metastasis, significantly correlating with low patient survival rates. Rapidly emerging evidence indicate that the extracellular matrix (ECM) composition and remodeling profoundly affect SSC metastatic dissemination. In this review, we will summarize the current knowledge on the role of ECM and its remodeling enzymes in affecting the growth and dissemination of UADT SCC. Taken together, these published evidence suggest that a thorough analysis of the ECM composition in the UADT SCC microenvironment may help disclosing the mechanism of resistance to the treatments and help defining possible targets for clinical intervention.

## 1. Introduction

Given their similar etiology and clinical features, esophageal and head/neck cancers are classified as tumors of the upper aerodigestive tract (UADT) [1]. UADT tumors are among the ten most common cancers worldwide and account for one million new cases diagnosed every year, of which approximately 90% are squamous cell carcinomas [1].The outermost layers of the nasal-oral cavity and esophagus are represented by stratified squamous epithelium (SSE), consisting of squamous epithelial cells layered on top of the basement membrane (BM), a thin specialized sheet of extracellular matrix (ECM). The SSE is constantly exposed to various stimuli, including harmful stresses. Indeed, the main risk factors for UADT SCC are represented by the use of tobacco and alcohol, betel quid chewing, and chronic mucosal irritation [2,3,4]. The exposure to accumulating carcinogens can also result in field cancerization leading to the occurrence of synchronous and metachronous malignancies in the entire UADT [5,6]. Another well characterized risk factor for these types of tumor is human papillomavirus (HPV) infection, which associates with poor response to the treatments and decreased overall survival of the patients [7,8,9]. Other causes include inherited gene defects in particular involving the CDKN2A locus and leading to the development of head and neck squamous cell carcinomas (HNSCC) [10].

Despite the continuous improvements in diagnosis and treatment, UADT SCC is still characterized by poor patients prognosis and low survival rate [1,11]. Therapeutic strategies for UADT SCC vary based on the stage and subtype of the disease and include surgery, chemotherapy, and radiation therapy [12,13,14].At present, surgical removal of the tumor remains the most commonly used treatment of esophageal SCC (ESCC) and oral SCC (OSCC), accounting for two-thirds of HNSCC [15,16]. A combination with preoperative neoadjuvant or postoperative adjuvant radiotherapy and chemotherapy has slightly improved patient prognosis [17,18,19]. The use of immunotherapy in combination with conventional chemotherapy has recently been considered as a possibility to significantly improve the outcome of these patients [12,20]. Despite the fact that these therapeutic approaches have given some benefits, the overall outcome of UADT SCC patients is still unsatisfactory. This is mostly due to the fact that these tumors are predominantly diagnosed at advanced stages, characterized by local or distant metastasis [1]. Therefore, unraveling the mechanisms of UADT SCC metastatic dissemination represents an important route for the development of more efficacious treatments.

Accumulating evidence indicate that microenvironmental, tumor cell-extrinsic, factors such as cytokines, chemokines, and proteins produced by tumor cells, as well as inflammatory or stromal cells, are also integral to UADT SCC growth and dissemination [21,22,23]. The tumor microenvironment (TME), comprising the tumor vasculature, the connective tissue, the infiltrating immune cells, and ECM, plays a key role in modulating cancer cell viability and proliferation, as well as the invasion and metastatic dissemination in many cancer types [24,25,26]. The ECM is a complex non-cellular compartment that provides physical scaffolding, and biochemical and biomechanical signals regulating tissue development and homeostasis. ECM dysregulation promotes the establishment of several pathologies including connective tissue disorders, muscular dystrophy, fibrosis, and cancer [27]. The concept that the ECM plays a relevant role in cancer has strongly increased over the last years. The ECM impacts on cancer growth and development both directly influencing tumor cell viability and motility, and indirectly modulating angiogenesis and tumor-associated inflammation [28,29]. Moreover, the altered deposition of ECM components affects the mechanical properties of the tumor and, as a consequence, their growth and progression. Additionally, its remodeling leads to the release of ECM-bound growth factors and ECM-fragments which profoundly influence the malignant behavior of cancer cells.

In this review, we will provide a brief overview of the routes exploited by UADT SCC cells during metastatic spreading and subsequently describe the current knowledge on the role of ECM in regulating HNSCC and ESCC metastatic dissemination. Finally, we will discuss the value of these microenvironmental cues as prognostic and predictive biomarkers and the putative impact towards the development of more efficacious anti-cancer therapies.

## 2. Metastatic Dissemination Routes of UADT SCC

**Figure 1:**
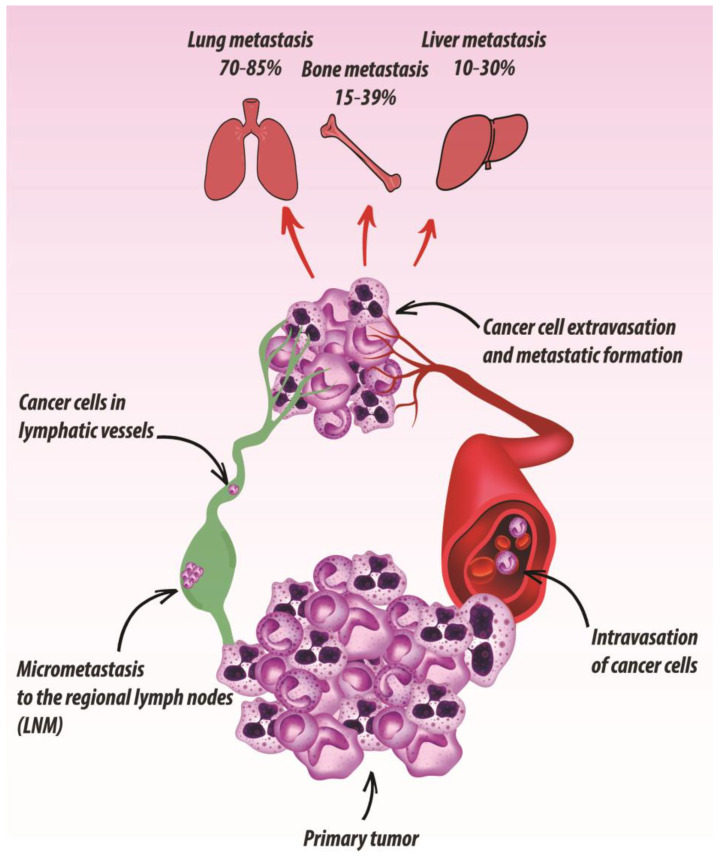
Schematic drawing of the metastatic spreading of UADT SCC.SCC tumor cells from the primary site penetrate the lymphatic vessels thus migrating to the regional lymph nodes forming Lymph Node Metastasis (LNM). Metastasis of SCC cells to the distant organs occurs both through the lymphatic and blood vessels mainly leading to lung, bone and liver colonization.

The dissemination of UADT SCC cells results in impaired therapeutic efficacy and poor patient outcome [30,31,32,33,34]. In patients diagnosed with advanced HNSCC and ESCC, the invasion of the surrounding tissues associates with loco-regional lymph node involvement as well as distant metastasis (Figure 1). Upon UADT SCC diagnosis, different clinical traits can be observed, ranging from single metastatic sites and controlled local disease, to widely disseminated metastasis [9,10,11].

The process of metastatic UADT SCC cell dissemination, as for other solid tumors, is complex and involves several steps [35,36], starting from the detachment of cells from the primary tumor. As a first event, SCC cells undergo epithelial-mesenchymal transition (EMT), resulting in reduced intercellular adhesion and increased cancer cell invasiveness [37,38,39,40]. Many intracellular molecules belonging to the Wnt, Notch, mitogen-activated protein kinase (MAPK), as well as the protein kinase B (AKT)/extracellular signal-regulated kinase (ERK) pathways orchestrate this process. The loss of E-cadherin and the concomitant increase of expression of mesenchymal vimentin and N-cadherin, promote cell elongation and derange cell polarity [41]. As a consequence, cancer cells locally breach the BM to invade the surrounding ECM and connective tissues. Subsequently, tumor cells reach the lymphatic and/or blood vessels and travel to distant metastatic sites. Cells that succeed in surviving in the blood or lymphatic circulation harsh conditions extravasate into the stroma, colonizing the metastatic site [36]. The pre-metastatic niche is established before the arrival of tumor cells from the primary tumor and facilitates the seeding of malignant cells [42]. Its formation is mediated by secreted factors deriving from the primary tumor, which mainly act recruiting immune cells and inducing ECM remodeling due to the increased activity of lysis oxidase (LOX) and metalloproteases [42,43,44].

### 2.1. Regional Metastasis

Regional nodal metastasis arise once tumor cells, at the primary HNSCC site, penetrate the lymphatic channels and migrate to the regional lymph nodes of the neck, thus forming micrometastasis [45]. Lymph node metastasis (LNM) are critical prognostic indicators for oral and oropharyngeal carcinomas [46]. The spread to regional lymph nodes is made possible by the highly invasive nature of OSCC cells and the sustained lymphatic drainage from the oral cavity. The most common sites for OSCC metastasis are the cervical lymph nodes, and once established, the survival rate of the patients is reduced by 50% [47]. Cancer cells usually spread to the lymph nodes on the same side of the cancer primary site. However, controlateral or bilateral lymph node metastasis can occur, albeit rarely [47].

Among the various clinical risk factors associated with ESCC pathogenesis, lymph node metastasis significantly contribute to poor prognosis, with the overall 5-year survival rates post-surgery dropping from 70–92% to 18–47% in patients with LNM [48]. Hence, the accurate identification of the LNM status, by imaging and molecular approaches, plays a crucial role in determining treatment strategies as well as prognostic outcomes [49]. In ESCC, the colonization of lymph nodes by metastatic cells is dependent on the primary tumor site, the T-stage, and the tumor histotype [50]. Furthermore, since neo-adjuvant chemoradiation treatments affect not only the frequency but also the localization of nodal metastasis, it is important to take this into account in the subsequent radiotherapy and surgical approaches [51,52]. For upper, middle, as well as lower thoracic esophageal SCC, the stations around the esophagus are among those with the highest prevalence of lymph node metastasis. A multidirectional spread of lymph node metastasis in the abdomen, the mediastinum, and the neck is determined by the presence of a dense lymphatic network surrounding the esophagus [50]. Additionally, ‘skip metastasis’, skipping the first and directly metastasizing into the second or third lymph node echelons, are frequently seen. This contributes to the presence of lymph node metastasis at unexpected distant sites, which makes it difficult to standardize the extent of the radiation field and lymphadenectomy [50].

### 2.2. Distant Metastasis

At diagnosis, distant HNSCC metastasis are present in about 10% of the cases with an additional 20–30% developing metastasis during the course of the disease [47,53]. Diagnosis of distant metastasis is associated with unfavorable prognosis, with a median survival rate of about 10 months [54]. Positive regional lymph node involvement, extracapsular invasion of tumor cells, and HPV negativity are key factors increasing the risk of primary tumor cell dissemination to distant organs [55]. The lung is the commonest site for HNSCC metastatization, accounting for approximately 70–85% of the cases, followed by the bone, about 15–39% cases, and the liver, accounting for 10–30% of the cases. Other poorly described metastatic sites include skin, mediastinum and bone marrow [53].

The impact of ESCC distal metastasis on the survival and outcome of the patients has been widely investigated in various studies [33,56,57,58,59]. The prognosis of ESCC patients with distant metastasis is very poor, with a 5-year survival rate <5%. In recent years, the use of inhibitors of PD-L1 (pembrolizumab), VEGFR2 (ramucirumab), and HER-2 (trastuzumab) has significantly improved the overall 5-year survival rate [13,60]. Nonetheless, the establishment of an optimal treatment for ESCC with distant metastasis requires further studies and clinical trials. The most common distant metastasis sites of ESCC are, in descending order, the lung, the liver, and the bone [60,61]. In rare cases (1–5%) brain metastasis are observed in esophageal cancer patients [41]. Interestingly, ESCC shows a peculiar tendency for unexpected specific metastatic sites, such as the skin, penis, lips, or retina [62]. This is due to the fact that distant metastasis can leave the esophagus not only via lymphatic and venal routs, but also through the arteries which are numerous in this district [62].

In recent years, the relevance of the microenvironment in metastatic dissemination has been increasingly recognized, and besides tumor cell-intrinsic factors, much attention is now focused on stromal factors, ECM, and ECM remodeling [63].

## 3. ECM as a Multi-Armed Warrior in SCC Dissemination

The ECM exerts a strong impact on all the TME components. For their structural features, ECM molecules can interact with a variety of proteins, receptors, and soluble factors, thus influencing a plethora of signaling pathways involved in multiple processes, such as EMT, angiogenesis, lymphangiogenesis, as well as resistance to the therapies [64].

Through the engagement of cell surface receptors, interaction with other ECM molecules and release of growth factors/cytokines upon remodeling, the ECM significantly influences the behavior of tumor cells, as well as other tumor-associated cell types such as infiltrating leukocytes, vascular endothelial cells, pericytes, and lymphatic endothelial cells [28,65,66,67].

**Figure 2:**
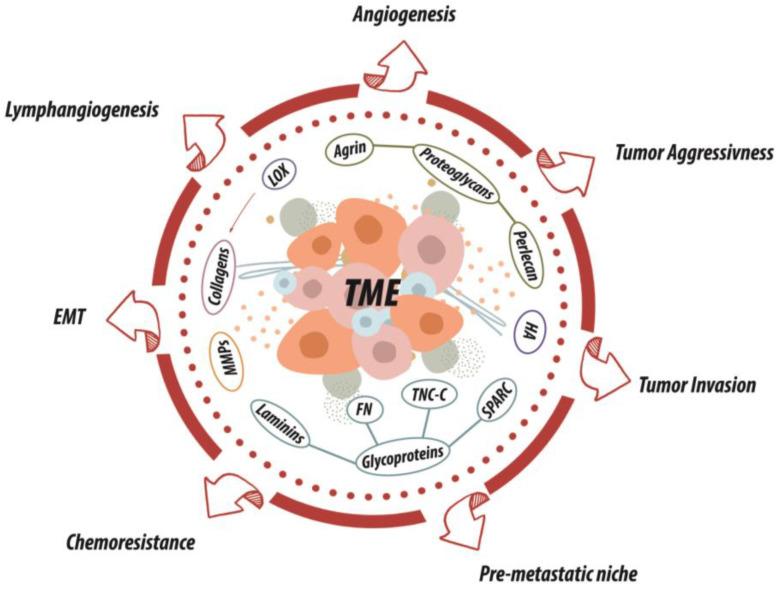
Schematic representation of the major ECM molecules affecting multiple aspects of UADT SCC development. TheECM molecules interact with different proteins, growth factors and receptors impacting on several processes determining the fate of the SCC, such as: angiogenesis, lymphangiogenesis, tumor aggressiveness and invasion, pre-metastatic niche establishment, chemoresistance, and epithelial-mesenchymal transition (EMT).

The reciprocal interactions occurring between cancer cells and the surrounding ECM orchestrate a complex cascade of events during USDT SCC malignant transformation (Figure 2). This continuous crosstalk impacts on many processes determining the tumor cell fate. In SCC, the ECM molecules play a direct role starting from the early phases of tumor formation, affecting the conversion of premalignant to malignant lesions [68], modulating the EMT processes, as well as influencing the invasive potential of SCC cells [68,69].

Integrins are the main receptors mediating the outside-in signals deriving from the ECM macromolecules, thus allowing the tumor cells to sense and react to the surrounding TME-derived stimuli [70,71]. Functioning as a link between the cytoskeleton and the extracellular environment, integrins activate signaling pathways controlling cell growth, differentiation, migration, and invasion [72]. Among these pathways, the mitogen-activated protein kinase-extracellular signal-regulated kinase (MEK-ERK) and phosphoinositide 3-kinase-protein kinase B (PI3K/Akt) signaling pathways are up-regulated in the presence of the ECM molecules [73]. Notably, changes in ECM composition and integrin profiles can exert profound effects on UADT SCC progression. Indeed, SCC tumors often display an altered expression of many integrins, impacting the activation of the ERK/MAPK signaling pathways. As an example, the T188I mutation of β_1_ integrin results in sustained ERK activation, whereas the up-regulation of integrin α_5_ associates with altered PI3K/Akt activation [74,75]. In OSCC, the major integrin receptors endowed with prognostic value include α_2_β_1_, α_3_β_1_, α_5_β_1_, and α_6_β_4_ [76]. The distinct expression of the integrins α_6_β_4_ and α_6_β_1_ at the invasion front, as well as the maintenance of a polarized integrin expression pattern in the tumor tissue, may serve as valuable new markers to assess ESCC aggressiveness [76]. In these patients, the α_5_ subunit plays an important role in the progression of the metastatic disease and represents a novel biomarker to predict the prognosis of ESCC patients [77]. Integrins are also emerging as promising mediators of lymph node metastasis since they mediate the interaction between metastatic cells and the lymph node-associated ECM, mainly composed by laminin, collagens, fibronectin, and vitronectin [78].

The adhesion of transformed cells to the ECM triggers outside-in signals which induce the expression and activation of catalytic enzymes, such as matrix-metalloproteinases (MMPs), that promote ECM remodeling and, in turn, cause the release of growth factors and active fragments. Among all the cytokines, the ECM processing results in the release of a number of key factors such as vascular endothelial growth factors (VEGFs), fibroblast growth factor (FGF), and hepatocyte growth factor (HGF) [28]. These molecules play a crucial role in tumor progression, as they are main drivers of the lymphangiogenesis and angiogenesis [79,80]. Indeed, one of the most important aspects of metastatic dissemination is the presence of newly formed lymphatic and blood vessels within the tumors. As mentioned above, these vessels represent the pathways through which tumor cells reach local or distant organs and establish new metastatic foci. In fact, the presence tumor-associated lymphangiogenesis is an indicator of the risk of lymph node metastasis in patients with UADT SCC [81,82]. In these patients, the increase of lymphatic vascularization leads to lymphatic invasion and subsequent lymph node metastasis [82]. Tumor-induced lymphangiogenesis is mediated mostly by VEGF-C and VEGF-D, produced and secreted by the tumor cells themselves, stromal cells, tumor-infiltrating macrophages, or activated platelets [83]. The high expression of VEGF-C and VEGF-D, as well as of other cytokines, including angiopoietins, insulin-like growth factor, and fibroblast growth factor, is associated with lymph node metastasis and poor prognosis in HNSCC [84]. VEGF-A, another member of VEGF family, is the main cytokine prompting tumor angiogenesis [85]. The development of new blood vessels is required to supply the tumor cells with nutrients and oxygen and occurs when the balance between pro- and anti-angiogenic factors tilts towards a pro-angiogenic TME. However, tumor associated vessels are abnormally leaky and represent a permissive route for metastatic dissemination. In UADT SCC, a high expression and activity of pro-angiogenic factors such as VEGF-A, HGF and FGF correlates with a more advanced disease and poor patient prognosis [86].

Lastly, increasing evidence indicate that ECM can dramatically influence the therapeutic response to the treatments impacting on the patient outcome. The increased ECM stiffness associated with tumors results in elevated interstitial pressure which acts as a barrier inhibiting the penetration and diffusion of the therapeutic drugs [87]. Impaired drug delivery can also be exacerbated by the formation of non-proficient vasculature, when the developed vessels are leaky and inefficient in delivering the therapeutics to the tumor site [88]. Some ECM molecules have been shown to play a key role in this context by impinging on VEGF-A and interleukin 8 (IL-8) pathways in other tumor types, i.e., in melanoma [89,90]. Given the important role of VEGF-A and IL-8 in determining UADT SCC outcome, we can hypothesize that the same mechanisms related to drug delivery and efficacy may take place also in HNSCC and ESCC. An additional mechanism through which the ECM influences USDT SCC chemoresistance relies on the interaction between integrins, in particular integrin α_5_, and the ECM which activates the FAK/PI3K/AKT signaling cascade protecting cells from the drug-induced apoptosis [91,92].

Understanding how the ECM composition and biomechanical properties affect cancer progression and the response to chemotherapeutic drugs is vital towards the development of targeted treatments.

## 4. ECM in UADT SCC: An Intertwined Story

**Figure 3:**
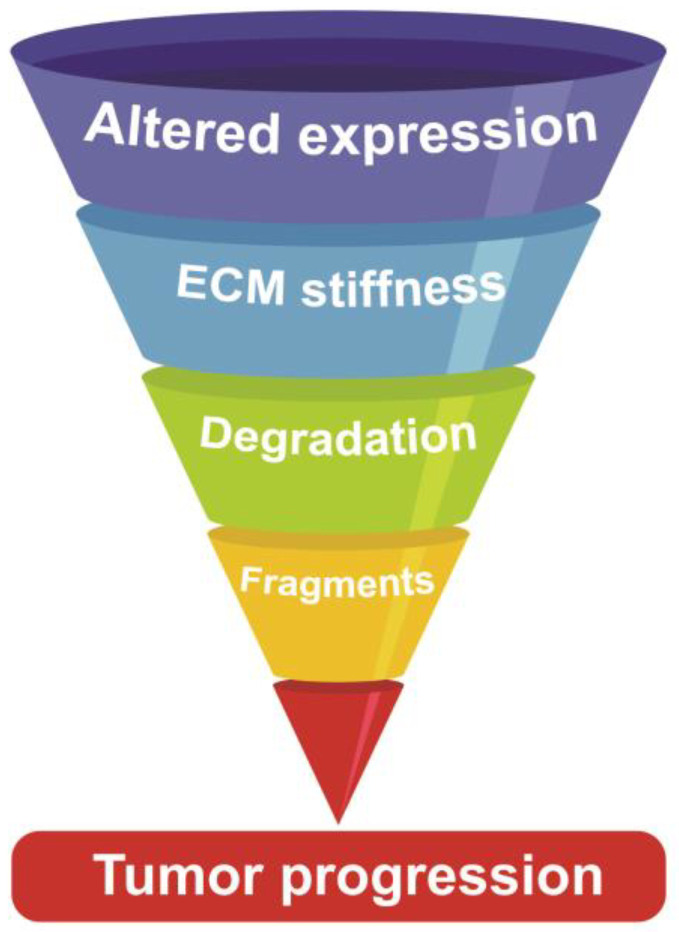
Schematic representation of the mechanisms by which the ECM affects tumor progression. In the TME, the ECM composition is modified due to altered expression of ECM molecules, mainly mediated by CAFs, and to the formation of inter- and intra-molecular crosslinking resulting in increased tissue stiffness. Furthermore, the higher levels of proteolytic enzymes lead to increased ECM degradation and the release of biologically active fragments/growth factors.

The ECM can be divided into two specialized types of matrices: the BM and the interstitial matrix (IM) [93]. Under healthy conditions, the BM is a well-structured ECM-composed sheet underlining epithelial and endothelial cells and separating them from the IM. The IM makes up for most of the stroma and plays a major role in cell migration, cell adhesion, angiogenesis, tissue development and repair. In the TME, the ECM composition is utterly abnormal due to different processes: first, increased expression of ECM molecules by cancer-associated fibroblast (CAFs) [94,95,96], which in conjunction with higher levels of modification enzymes, contributes to increase tissue stiffness [97,98]; second, a concomitant activation of proteases leading to ECM degradation and remodeling, with a consequent release of active fragments and growth factors [99,100] (Figure 3).

During tumor progression, despite recent evidence indicating that tumors cells may also play a role, CAFs are the major culprits for the dysregulated collagen turnover leading to fibrosis, characterized by excessive collagen depositions [101,102]. CAFs display distinct morphological and biological characteristics from normal fibroblasts. From the molecular point of view, they express high levels of α-smooth muscle actin (α-SMA), fibroblast activation protein (FAP), fibroblast-specific protein-1 (FSP-1), platelet-derived growth factor receptor α/β (PDGFR α/β) and vimentin [22].CAFs promote UADT SCC progression by secreting cytokines/growth factors and ECM proteins, as well as MMPs, thus influencing tumor cell proliferation, angiogenesis, inflammation, metastatic invasion, and drug resistance [103,104,105,106].

The ECM comprises two classes of macromolecules: fibrillar proteins and proteoglycans. Fibrillar proteins such as collagens, fibronectin, and laminins display both structural and functional properties. Not only do they represent a scaffold for the cells, but also induce cellular responses following the engagement of cell surface receptors. The main ECM molecules exerting a role in UADT SCC are detailed in the following paragraphs and summarized in Table 1.

### 4.1. Collagens

Collagens are the most represented proteins in the ECM [99,152] and are organized in a meshwork surrounded by proteins such as elastin and glycoproteins causing resilience to the extensive tensile strength. Twenty-eight different collagens have been so far identified, thus providing a unique ECM composition in different tissues. In the BM, the most represented collagens are type IV and type VIII, whereas the IM is prevalently composed by type I, II, III, V, XI, XXIV, XXVII collagens. Type VI collagen is found in the interface between the BM and the IM. Even if many of these collagens are mis-regulated in UADT SCC cancer, among them collagen I is the most extensively studied and its expression is often increased in this tumor type [105,107]. Collagen I harbors two CYP1 chains (COL1A1) and a single CYP2 chain (COL1A2). In ESCC and OSCC, through the engagement of integrin α_v_β_8_, the COL1A2 chain promotes EMT by activating the FAK/MEK/ERK signaling pathway resulting in higher tumor cell aggressiveness [107,108]. Of note, the expression of COL1A1 was one of the highest among other collagens analyzed [102]. In the same study, and in accordance with other publications [101,153], it was reported that the mis-regulation of most of the collagen genes is a common trait in USDT SCC. Among them, the expression of COL1A1, COL10A1, and COL11A1 were found to be particularly high in tumor tissues compared with normal counterpart; on the contrary, the expression of COL4A4, COL6A5 and COL14A1 was significantly lower in tumor tissues. Importantly, the expression of COL6A5 and COL18A1 strongly correlated with the overall survival of ESCC patients and might represent a robust prognostic biomarkers for ESCC [102].

The collagen content in the TME is the result of the balance between gene expression and proteolytic degradation, mostly due to MMPs hyper-activation. Notably, the cleavage of collagens can lead to the release of biologically active fragments that, in turn, can influence other processes within the TME [99]. As an example, the C-terminus of collagen I, once released, induces the expression of cytokines such as VEGF-A as well as MMP-2 and -9, further promoting ECM remodeling and angiogenesis [154]. The collagen XVIII derived C-terminal fragment endostatin is one of the most important soluble factors stimulating tumor angiogenesis [155]. In UADT SCC, endostatin displays a key role in vessel formation and tumor dissemination, also influencing the efficacy of targeted and radio therapies; for these reasons endostatin is under evaluation as valuable target for combinatorial therapeutic approaches [156,157].

Collagens can trigger intracellular signaling also in an integrin-independent manner. For instance COL11A1 through the engagement of the receptor tyrosine kinase discoidin domain receptor 1 (DDR1) stimulates the proliferation and migration of HNSCC cells and attenuates the apoptotic response to cisplatin [109]. Once activated, DDR1 triggers a number of downstream signaling pathways [110], inducing the expression of pro-inflammatory mediators as well as matrix degrading enzymes overall resulting in more migratory and invasive phenotypes [158]. In HNSCC, COL11A1 has been implicated in the formation of lymph node metastasis, since its expression was shown to be seven-fold higher compared with tumors that had not metastasized [111,112].

### 4.2. Fibronectin

Another fibrillar ECM molecule affecting UADT SCC dissemination is fibronectin (FN) [159,160,161]. As opposed to plasma FN, the type of FNs up-regulated in tumors, referred to as oncofetal variants, harbor alternatively spliced exons encoding the highly conserved FN type III extra domains A (EDA) and/or B (EDB). Notably, FN-EDA is a marker of the tumor vasculature [162] and a principal component of the pre-metastatic niche in a variety of tumors [113]. In both HNSCC and ESCC, FN expression is increased compared with normal tissues and its high expression associates with poor patient prognosis and resistance to radiotherapy [114,115,116]. A high FN content facilitates tumor cell metastasis by promoting morphological changes and improving the motility and migratory ability of ESCC cells. In this context, FN acts as a physical scaffold laying the path for tumor cell invasion. The migration of SCC cells on fibrillar FN-rich matrices is achieved through the engagement of integrins α_v_β_6_ and α_9_β_1_ [115], and associates with the activation of latent TGF-β at the tumor-stroma interface which, in turn, can further support tumor progression by prompting angiogenesis [117]. In case of highly invasive OSCC cells expressing low levels of E-cadherin, FN induces fast cell migration associated with increased Rac1 activation and weaker cell-ECM adhesion; on the other hand, in high E-cadherin-expressing poorly invasive OSCC cells, FN produces a collective, non-directional migration, with high RhoA activity and altered cell-ECM adhesion [118].

### 4.3. Laminins

Together with collagen IV, laminins are the major components of the BM [163]. Laminins are composed by a combination of α, β and γ chains and exert a number of effects on adjacent cells, including cell adhesion, cell migration and cell differentiation, mainly occurring via integrin engagement [164]. Their importance in BM homeostasis is highlighted by the fact that, during tumor progression, laminins are considered a molecular marker of BM degradation. The deposition of a number of laminins is altered in the UADT SCC TME [165], and their expression level can be useful to evaluate the histological differentiation and aggressiveness of some HNSCC [166].

The most studied laminin in the context of UADT SCC is laminin-5 (also known as laminin 332) an epithelial-BM-specific variant [119]. Its heterotrimer is composed of the α3, β3, and γ2 chains, encoded by the LAMA3, LAMB3, and LAMC2 genes, respectively. Laminin-5 promotes cell survival, proliferation, and migration by triggering the activation of integrin α_3_β_1_ and α_6_β_4_ and the downstream phosphatidylinositol 3-kinase (PI3K) [120].

In OSCC, laminin-5 is over-expressed and its increased levels associate with enhanced tumor invasiveness [119]. Interestingly, alterations are observed not only in the amount but also in the deposition pattern, which shows peculiar irregularities [121]. Invading cells adhere to the aberrant laminin-5 structure and migrate through the interaction with integrin α_3_β_1_. Highly invasive OSCC cells show an increased motility on laminin-5, when compared with less invasive cell lines [122]. This increased motility is thought to be regulated by the enhanced integrin α_2_β_1_ and α_3_β_1_ expression [167]. The same integrin engagement by tumor cells occurs under conditions consistent with lymphodynamic flow. These interactions are supposed to be critical for downstream tumor cell growth and survival within the lymph node microenvironment [78]. The finding that in HNSCC the laminin-5/α_6_β_4_ integrin binding is targeted by *miR-29s*, leading to decrease cell invasion, allowed to speculate for new potential therapeutic strategies for these patients [123].

### 4.4. Tenascin-C

Tenascin-C (TNC) is a hexameric, multimodular ECM protein with several molecular structures generated through alternative splicing and protein modifications [168]. TNC has many binding partners, including other ECM molecules, cell surface receptors, and soluble factors [169]. TNC over-expression is repeatedly observed in cancer, often at the invasive tumor front [170], and associates with poor clinical outcome in several malignancies, including UADT SCC [124,125,126]. Due to the multiple interactions in which it is involved, TNC directs a plethora of cell signaling and gene expression programs, thus shaping mechanical and biochemical traits within the TME [171]. TNC exerts pro-tumorigenic functions interacting with a variety of cell types within the TME, including cancer cells themselves, CAFs, lymphocytes, and tumor-associated macrophages, as well as endothelial cells thus promoting angiogenesis [172]. TNC, together with other ECM molecules such as laminin-5 [127], takes part in the formation of a meshwork functioning as a route for cancer cell invasion, additionally it also stimulates tumor cell invasion by promoting the EMT switch via the Akt/HIF1α axis [128]. Interestingly, it was recently shown that TNC contributes to the formation of an immune-suppressive lymphoid stroma activating the CCL21/CCR7 signaling pathway [129]. The subsequent increased recruitment of T regulatory cells and the enhancement of the expression of anti-inflammatory cytokines further contributes to the pro-metastatic effects associated with high TNC expression levels.

Interestingly, compared to the healthy controls, higher TNC serum levels are detectable in patients affected by late-stage HNSCC or recurrent disease [130]. Even if further investigations will be necessary to better evaluate if the serum levels of TNC hold value as tumor markers, these results grant further hope for the development of ECM-derived markers useful to predict the clinical outcome of patients.

### 4.5. SPARC

The secreted protein acidic and rich in cysteine (SPARC) was originally identified as a collagen-binding glycoprotein and it is involved in many biological processes, including tissue remodeling, angiogenesis, and cancer cell differentiation and migration [173]. The functions of SPARC are mediated by multiple interactions with MMPs and growth factors, endowing the molecule with the capability to evoke a number of events within the TME. The role of SPARC in carcinogenesis is controversial and context dependent. In melanomas and gliomas, enhanced SPARC expression associates with a highly aggressive tumor phenotype, whereas in pancreatic adenocarcinoma, acute myeloid leukemia, and ovarian and colorectal carcinomas, SPARC behaves as a tumor suppressive molecule [174,175,176,177]. These opposing actions of SPARC may be due to the peculiar molecular mechanisms characterizing the various tumor types, as well as by the differential expression of cancer-specific proteases [173].

Many investigations report that in UADT SCC the SPARC expression level is higher compared to the normal tissue. In HNSCC, SPARC represents a powerful independent prognostic marker for short disease-free interval and poor overall survival [131]. The same role for the molecule was demonstrated in ESCC, in which high SPARC expression closely associates with ESCC metastasis [132]. Overall, the level of SPARC in the UADT SCC TME represents a potential predictor of poor prognosis and has been shown also to associate with impaired sensitivity to chemotherapy [133]. All these evidence prompted to propose SPARC as a therapeutic target for these types of tumors [134].

### 4.6. Proteoglycans

In the context of UADT SCC, there are few published evidence demonstrating a prominent role of proteoglycans in determining their fate. However, most likely this does not mean that proteoglycans do not play a role in this context. In fact these type of molecules have been shown to impact tumor growth and development through different processes [28,178,179,180], and the lack of knowledge should prompt to verify the role of these molecules also in UADT SCC.

Among the main heparan sulfate proteoglycans (HSPG), agrin and perlecan [181] are important components of the BM and are over-expressed in some cancers, such as prostate cancer, hepatocellular carcinoma, and breast cancer, and their higher levels associate with tumor development and progression. Interestingly, these HSPGs also affect UADT SCC development [182].

Perlecan consists of a protein core, divided into several unique structural regions, modified by the addition of N-terminal heparan sulfate side chains, each imparting distinct functional diversity to the molecule [183,184]. In OSCC, perlecan is differentially expressed and its functions are highly dependent on the peculiar invasive properties of the tumor [135], indicating that its role is dependent on other TME components, such as the expression of growth factors which are retained within its meshwork, as well as the activity of specific proteases responsible of its processing. In this view, the proteolytic cleavage of perlecan by cathepsin L results in the generation of a C- terminal fragment named endorepellin which exerts its own biological activity [185,186]. No data are available regarding the role of endorepellin in UADT SCC, however, since cathepsin L is over-expressed in this context [136], we can envision that endorepellin may also impinge on these tumor types. It is interesting to note that, while perlecan exerts strong pro-angiogenic functions, its fragment endorepellin displays opposite effects. The fine regulation of angiogenesis by these two players should be envisioned as an additional mechanism impacting on UADT SCC metastatization.

Agrin shares with perlecan a rather intriguing multimodular organization [187]. The amino acid sequence of agrin encodes for a protein with a molecular size of 220 kDa, however the apparent molecular weight in SDS-PAGE is around 400 kDa due to the presence of long heparan sulfate and chondroitin sulfate glycosaminoglycans (GAGs) chains attached to the protein core [182]. In hepatocellular carcinoma, agrin acts as a sensor boosting oncogenic signals and regulating Arp2/3-dependent ruffling, invadopodia formation, and EMT through sustained focal adhesion integrity [137]. In UADT SCC, high agrin expression is predictive of poor patient prognosis [187]. Agrin influences tumor cell migration, adhesion and resistance to chemotherapy impacting on FAK, ERK and cyclin D1 activation [187]. Upon local invasion processes, agrin is processed by MMP-3 and neurotrypsin, generating a C-terminal biologically active soluble fragment [138]. Importantly, this fragment could represent a promising new biomarker for pathological processes, including sarcopenia, renal dysfunction and colorectal cancer [139,140,141]. If this fragment can function as a potential marker also in UADT SCC, needs to be determined.

### 4.7. Hyaluronan

Hyaluronan (HA), a non-sulphated glycosaminoglycan, is another ECM component with multiple functions in healthy conditions as well as in disease, including cancer [188,189,190]. In UADT SCC, HA is often altered and its increase associates with a higher tumor invasive phenotype [142,143,144]. During tumorigenesis, HA is processed in small fragments by different hyaluronidases among which Hyal-1 is the most abundant in HNSCC; since these fragments are detectable in the saliva, HA might serve as an early easily detectable marker for HNSCC [145,146,147]. The major cell-surface receptor for HA is CD44; its expression is increased in the sites of tumor invasion and one of its isoforms, CD-44v3, has been closely linked to progression and to chemoresistance of UADT SCC [191]. The interaction of HA with CD44 has been shown to be key in regulating the SCC stem cell signaling cascades [192,193,194]. Though the role of the HA-CD44 signaling axis has not been fully unveiled in these types of tumors, it has been demonstrated that this interaction promotes a complex Nanog-Stat3 signaling pathway that regulates the miR-21 gene expression and production. As a consequence, miR-21 down-regulates the tumor suppressor protein programmed cell death 4 (PDCD4), leading to HNSCC cell survival and chemoresistance [148]. Beside miR-21 activity, the expression of miR-302 was also induced in HNSCC following the interaction of HA with CD44 via the formation of the OCTA4/SOX2/Nanog complex [149]. In turn, enhanced miR-302 expression favors tumor cell survival and chemoresistance [148,149,195,196,197].

Additionally, HA has been demonstrated to promote HNSCC progression by promoting the association of CD44 with EGFR with a subsequent enhanced activation of EGFR and the downstream MAPK pathway, one of the most altered pathways in HNSCC [150]. The phosphorylation of EGFR prompted by the interaction between HA and CD44 has been shown to regulate ERK 1 and ERK 2, key in driving tumor cell proliferation and migration [150,151].

Taken together, these evidence have shed some light into the understanding of the molecular mechanism by which HA affects head and neck cancers, opening the possibility to develop new drug targets but also to exploit its fragments as early markers for HNSCC.

## 5. EMC Stiffness: The Dark Side of the Mechanical Force

Although tumor stiffness has been mainly studied in other tumor types, biophysical forces display an important effect also in UADT SCC formation and development [198,199,200]. In general, tumor tissues are often characterized by pronounced stiffness of which the cross-linking of collagen is the major culprit [97]. In fact, increased stiffness derives from both a higher expression of collagens, mainly collagen I, and an increased activity of lysis oxidase (LOX) enzymes, which produce intermolecular cross-links between collagen I fibers themselves and other proteins such as collagen III and IV and FN [201,202]. Stiffness elicits behavioral effects on the adjacent tumor cells affecting cell proliferation, differentiation, as well as migration and invasion, thus impacting on the metastatic process [201,203]. Among all the tumor-associated cell types, endothelial cells are particularly sensitive ECM mechanical property changes. Mechanical strength alterations in the TME significantly impact endothelial cell signaling and behavior, triggering angiogenesis and consequently favoring tumor cell dissemination [204]. High tumor stiffness was shown to promote EMT in SCC cells and to enhance their motility [98]. The molecular mechanism driving EMT in this context relies on FAK (focal adhesion kinase) activation and PI3K/integrin signaling [205,206]. More in detail, the high matrix stiffness triggers integrin activation thus promoting the signaling through the FAK-Src complexes which consequently induce a Rho/ROCK-dependent myosin-mediated cellular contractile force, resulting in an invasive phenotype [207]. However, the stiffness sensitivity of cancer cells appears to be context dependent. Squamous carcinoma cells of the human tongue are less stiff compared to the healthy tissue, confirming that high stiffness is not always systematically associated with tumor phenotypes [208]. At the clinical level, increases in stiffness seem to correlate with advanced stages of the disease and shorter recurrence-free survival time. Taken together, these evidence suggest that the progression of oral cancers, similarly to other epithelial tumors, is mechanically sensitive [200].

## 6. The Turmoil of Scissor-Handed Proteases

ECM remodeling is an ongoing process functional to tissue development and repair as well as in pathological conditions as cancer [209]. The interweaving of ECM within the TME relies on the activity of proteases, whose mechanism of action has been thoroughly investigated in a variety of cancers [210]. Among them, MMPs are the major players [204].

MMPs are zinc metalloenzymes encoded by at least 26 distinct genes [100,211,212]. The members of the MMP family display structural similarities, albeit with substantial differences, however, they are all produced as inactive precursors harboring a pro-peptide sequence. The cleavage of the pro-domain results in MMP activation and occurs in the pericellular space in an integrin-dependent manner (e.g., for pro-MMP2) or intracellularly due to the activity of furin-like proprotein convertases (e.g., for MT-MMPs) [213]. The catalic domain possesses a zinc (Zn^2+^) active site linked to the hemopexin-like C-terminal portion [214,215], which determines the substrate specificity [216,217]. MMPs are capable of cleaving a number of ECM components, resulting in their degradation and, often, the release of active fragments, as for instance demonstrated for collagen XVIII, perlecan and agrin [135,138,154]. Since the ECM is a reservoir of many growth factors, the MMP activity induces the release of a number of cytokines such as VEGF-A, -C and -D, FGF, and EGF [204]. MMP function is tightly regulated at both transcriptional and post transcriptional levels by the action of pivotal growth factors, such as TGF-β1 [218], as well as their specific activating enzymes and/or inhibitors [219], respectively.

MMPs have been found to be up-regulated in many cancer types [220]. In the UADT SCC, MMPs play critical roles impacting key processes such as angiogenesis, local invasion, and tumor cell intravasation and extravasation, thus displaying a strong impact in metastasis formation [221]. The expression of multiple MMPs has been extensively investigated in UADT SCC, these analyses demonstrated that the expression of MMP-1, MMP-2, MMP-3, MMP-7,MMP-9, and MT1-MMP was significantly up-regulated compared to the normal tissues [87,222,223]. Importantly, the expression of MMP-2, -3 -7, -9 positively correlates with the depth of invasion, lymph node metastasis, and vessel permeation in these patients [223,224,225,226]. Not only the levels, but also the spatial localization of MMPs is important in determining their impact during tumor progression. Notably, the expression of MMP-7 at the invasive tumor front associates with the disease recurrence and with a shorter disease-free and overall survival [227]. On the contrary, increased MMP-1 levels in ESCC inversely correlate with the patient prognosis [228]. It is interesting to point out that MMP-1 is responsible for collagen degradation within the primary tumor site, thus playing a crucial role in local invasion, while its activity does not affect the distant dissemination of tumor cells [228]. Among the altered MMPs, strong attention has been paid to the potential prognostic role of MMP-9 given the crucial role of this MMP in EMT and tumor angiogenesis. Indeed, the knockdown of MMP-9 attenuates the TGF-β1-induced EMT preventing the invasiveness and migration of ESCC cells [229]. In fact, increased MMP-9 expression positively associates with the expression levels of vimentin and SNAI1. Notably, MMP-9 participates in the proteolysis of the microvascular BM and the subsequent intravasation of cancer cells, thus contributing to metastatic dissemination [227,229,230]. In accordance, due to its role in driving UADT SCC progression, MMP-9 up-regulation correlates with shortened relapse-free survival and poor patient prognosis [231,232].

Recently, the MMP-1 and MMP-9 plasma levels in UADT SCC patients have been considered as potential prognostic or predictive biomarkers. High plasma levels of free MMP1 have been shown to associate with a worse ESCC patient prognosis [233]. On the contrary, the MMP-9 plasma levels were lower in UADT SCC patients compared with healthy individuals, thus MMP-9 has been proposed as a potential prognostic biomarker of overall survival for the response to chemoradiotherapy [234,235]. Taken together, these results suggest that MMPs represent a promising mean for the development of new non-invasive liquid biopsy-based tools to improve the management of UADT SCC patients.

Another group of proteases involved in UADT SCC progression is the ADAM family, consisting of transmembrane secretory proteins arranged in multiple domains [236,237]. These proteases play an important role in basement membrane degradation, cell migration, and metastatic dissemination [238,239,240,241,242]. In particular, increased levels of ADAM 8, 9, 10, 12, 17, and 28 have been found in OSCC [243,244,245]. Specifically, ADAM 10 modulates the malignant behavior of oral keratinocytes via the interaction with integrin α_v_β_6_ and the over-expression of MMPs [246]. Moreover, high levels of ADAM 12 correlates with increased OSCC progression [237], whereas the ADAM 17 associates with nodal metastasis, local recurrence, and OSCC invasion [247].

The major proteases and growth factors involved in UADT SCC development displaying putative value as biomarkers are summarized in Table 2.

## 7. Conclusions and Future Perspectives

In the past few years, the use of immunotherapy has opened a new perspective in the management of cancer patients, with promising improvements also in the treatment of UADT SCC. We foresee that, in the future, the identification of efficacious combinatorial treatments may represent an encouraging strategy to improve the patient outcome. However, we consider that to further improve the management of UADT SCC patients, the search for new biomolecular targets and a better understanding of the molecular mechanisms involved in metastasis formation are key for the identification and treatment of UADT SCC patients with high risk of recurrence. In this perspective, due to its multiple function in the TME, the ECM may represent a vital field of investigation.

## Figures and Tables

**Table 1:** Main ECM molecules involved in UADT SCC and their roles in tumor progression.

ECM Molecule	Receptors	Intracellular Signaling	Clinical Relevance	Sample Type	Expression	References
Type I collagen	αvβ8 integrin	FAK-MEK/ERK	Increased tumor aggressiveness (biomarker)	RNA levels in tumor tissue	Increased	[101,102,107,108]
Collagen A1 (XI)	DDR1	Shp-2, Src, MAPK	Lymph nodes metastasis (biomarker)	RNA and protein levels in tumor tissue	Increased	[109,110,111,112]
Fibronectin (FN)	αvβ6 and α9β1 integrins	TGF-β	Poor patient prognosis, resistance to radiotherapy (biomarker)	RNA and protein levels in tumor tissue	Increased	[113,114,115,116,117,118]
Laminin 5	α3β1 and α6β4 integrins	PI3K/AKT/mTOR	Increased tumor invasiveness (biomarker)	Protein levels in tumor tissue	Increased	[78,119,120,121,122,123]
Tenascin-C	Integrins	Akt/HIF1α, CCL21/CCR7	Poor clinical outcomes (biomarker)	Protein levels in tumor tissue and plasma	Increased	[124,125,126,127,128,129,130]
SPARC	Integrins	MAPK, PI3K/AKT	Poor clinical outcome and metastatic disease (biomarker)	Protein and RNA levels in tumor tissue	Increased	[131,132,133,134]
Perlecan	Growth factors	MAPK, VEGF-VEGFR	Increased tumor Invasiveness (biomarker)	Protein levels in tumor tissue	Increased	[135,136]
Agrin	Lrp4, MuSK	FAK/ERK/cyclin D1	Poor prognosis and chemotherapy resistance (biomarker)	Protein levelsin tumor tissue	Increased	[137,138,139,140,141]
Hyaluronan	CD44	Nanog-STAT3 MAPK/ERK	Chemotherapy resistance, increased tumor invasiveness (biomarker and potential therapeutic target)	Protein levels in saliva and RNA levels in tumor tissue	Increased	[142,143,144,145,146,147,148,149,150,151]

**Table 2:** List of valuable non-ECM biomarkers for UADT SCC detection and their clinical relevance.

Biomarker	Profile	Clinical Relevance	References
MMP -2,-3,-7,-9	Up-regulated in UADT SCC patients	Depth of invasion, lymph node metastasis and vessel permeation	[87,222,223,224,225,226]
MMP-7	Up-regulated at the invasive front of the tumor	Short disease-free and overall survival	[227]
MMP-1	Up-regulated in tumors and responsible for collagen degradation	Local invasion	[228,233]
MMP-9	Up-regulated in tumors and association with vimentin and SNAI1 levels	Shortened relapse-free survival and poor prognosis of patients	[229,230,231,232,234,235]
VEGF-A	Increased release upon ECM remodeling	Advanced disease and poor prognosis	[86]
VEGF-C, -D	Increased release upon ECM remodeling	Lymph node metastasis and poor prognosis	[84,86]
TGF-β1	Increased expression in UADT SCC patients	Distant lymph nodes metastasis, low rate of survival and poor prognosis	[218,229]
FGF, HGF, EGF	Increased release upon ECM remodeling	Poor prognosis, advanced tumor stage	[86,204]
Endostatin	Induces VEGF-A and MMP-2,-9 expression promoting ECM remodeling and angiogenesis	Important role in tumor dissemination influencing the efficacy of targeted therapies	[156,157]
ADAM 12	Over-expressed in OSCC	Increased tumor progression	[237]
ADAM 17	Over-expressed in OSCC	Nodal metastasis, local recurrence and OSCC invasion	[247]

## Data Availability

Not applicable.
